# Effects of aerobic exercise combined with resistance training on body composition and metabolic health in children and adolescents with overweight or obesity: systematic review and meta-analysis

**DOI:** 10.3389/fpubh.2024.1409660

**Published:** 2024-08-09

**Authors:** Xuan Liu, Qiang Li, FuXiang Lu, Dongsheng Zhu

**Affiliations:** Department of Physical Education, Ocean University of China, Qingdao, Shandong, China

**Keywords:** aerobic exercise, resistance training, body composition, metabolic diseases, pediatric obesity

## Abstract

**Background:**

To systematically review the effects of aerobic exercise and resistance training on Metabolic Health in children and adolescents with overweight/obesity.

**Methods:**

Employing a retrieval strategy that combines subject terms and free terms, searches were conducted in the CNKI, WanFangData, VIP, PubMed, Web of Science, Embase, and Cochrane Library databases up to October 31, 2023.

**Results:**

A total of 29 studies involving 2,195 subjects were included. The combination of aerobic and resistance training significantly reduces body composition and metabolic health in children and adolescents with overweight or obesity, as evidenced by changes in various parameters (BMI, WC, FM, BF%, VO_2max_, TG, TC, HDL-C, LDL-C, HOMA-IR, FPG, INS). However, there were no significant differences observed in hs-CRP. Subgroup analyses further showed that changes in intervention measurement had a significant effect on the effectiveness of the intervention.

**Conclusion:**

Aerobic exercise combined with resistance training has a positive impact on the physical health of children and adolescents with overweight/obesity. The recommended exercise prescription is at least three sessions of more than 60 min per week for 12 weeks or more for better health benefits.

## 1 Introduction

The prevalence of obesity has witnessed a significant surge over the past five decades. According to the World Health Statistics (2020) published by the World Health Organization (WHO), more than 340 million children and adolescents aged 5–19 years were overweight or obese worldwide in 2016 (1). Childhood obesity not only elevates the likelihood of becoming obese, facing premature mortality, and disability later in life, but it can also lead to breathing difficulties, a higher chance of bone fractures, high blood pressure, early indicators of heart disease, issues with insulin sensitivity, and psychological health consequences (2).

Recent studies have found that regular participation in exercise not only improves self-efficacy and awareness of exercise in children with obesity but also has a role in controlling weight gain and the development of mental health in children with obesity (3). Physical activity stands as a potent avenue for ameliorating obesity in children and adolescents, with lasting implications for adult physical health.

The Evaluation of Physical Activity Levels of Children and Adolescents Aged 7–18 Years, published by the National Health Inspection Commission (4), states that children and adolescents should spend an average of no less than 70 min of cumulative moderate- and high-intensity physical activity per day, of which at least one session of moderate- and high-intensity physical activity lasting for 10 min or more per day should be carried out. Additionally, the guidelines advocate for aerobic-based physical activity complemented by resistance exercises targeting muscle strength and bone health, to be performed at least thrice weekly.

While numerous investigations have validated the efficacy of exercise in ameliorating obesity in children and adolescents, extant literature predominantly examines the impact of various exercise modalities on body composition in this demographic. It was confirmed that aerobic combined with resistance training was superior to aerobic or resistance training alone in terms of BMI, and BF% (5). Building upon these insights, this research aims to examine the impact of combining aerobic workouts with strength training on body composition and indicators of metabolic health in young individuals who are overweight or obese, with the aim of furnishing evidence-based exercise recommendations for combating obesity in this population.

## 2 Methods

The execution of this meta-analysis adhered to the guidelines defined by the Preferred Reporting Items for Systematic Reviews and Meta-Analyses (PRISMA) (6).

### 2.1 PICOS and literature search strategy

The search for keywords was conducted using the Population, Intervention, Comparison, Outcome, and Study design (PICOS) framework (Table 1). The study subjects included children and adolescents with overweight or obesity, with no restrictions on gender or nationality.

**Table 1:** PICOS chart.

Population	Children and adolescents with overweight or obesity
Intervention	Aerobic exercise and resistance exercise
Comparison	Conventional activity
Outcome	Body composition and metabolic health
Study design	Randomized controlled trial, RCT and non-randomized controlled trial, NRCT

A search was conducted across CNKI, WanFangData, VIP, PubMed, Web of Science, Embase, and Cochrane Library using a mix of controlled vocabulary and free-text terms. The focus was on studies about the effects of combined aerobic and resistance training on body composition and metabolic health in children and adolescents with overweight/obesity, covering literature up to October 31, 2023.The main keywords used were (aerobic exercise OR resistance training or physical activities OR training) AND (obesity^*^ OR overweight OR obese^*^) AND (child^*^ OR adolescent^*^).

### 2.2 Study inclusion and exclusion criteria

The criteria for inclusion are as follows: (1) The literature is a full-text literature in English and Chinese. (2) The test group was aerobic exercise combined with resistance training and the control group was conventional activity. (3) The types of studies encompassed both randomized and non-randomized controlled trials. (4) Study subjects consisted of children and adolescents who were overweight or obese (as judged by the World Health Organization or their respective nationally recognized criteria) with no exercise-related contraindications and no other health problems or complications. (5) Outcome measures included body mass index (BMI), fat mass (FM), body fat percentage (BF%), maximal oxygen uptake (VO_2max_), triglycerides (TG), total cholesterol (TC), high-density lipoprotein cholesterol (HDL-C), low-density lipoprotein cholesterol (LDL-C), homeostasis model assessment of insulin resistance (HOMA-IR), high-sensitivity C-reactive protein (hs-CRP), fasting plasma glucose (FPG), insulin (INS).

Participants were excluded based on the following conditions: (1) Inclusion of control groups comprising normal-weight children and adolescents. (2) Meta-analysis of the research literature. (3) Programs lacking a combination of aerobic and resistance training. (4) Repetitive publications or literature tangential to the subject matter. (5) Literature lacking original data or precluding the derivation of mean difference (MD) and standardized mean difference (SMD) for outcome indicators.

### 2.3 Date collection or data synthesis

Literature was meticulously screened, and data were gathered by two researchers in accordance with the research objectives and predetermined criteria. In case of disagreement, a third researcher adjudicated. After perusing the selected literature, the researchers recorded the information on a predefined data collection form, including basic information (author’s name, date of publication, title of the article), number of participants, method of intervention, and outcome indicators.

### 2.4 Risk of bias assessment

Randomized controlled trials: 2 researchers conducted a comprehensive risk of bias assessment of the 23 included papers according to the Cochrane Handbook.

Non-randomized controlled trials: The methodological index for non-randomized studies (MINORS) (7) was used to evaluate the quality of the 6 articles included in the study, and there were 12 indicators, the first 8 of which were for studies without a control group, with a maximum score of 16 points, and the maximum score for studies with a control group was 24 points. The 12 indicators were evaluated together, with a total maximum score of 24; scores of 0–8 were classified as low quality, 9–16 as moderate quality, and 17–24 as high quality. The MINORS assessment framework stipulated that literature scoring below 12 points should be excluded from meta-analysis. The scoring process was conducted independently by two researchers, and in cases of discordance, resolution was achieved through deliberation or consultation with a third party, ultimately culminating in a consensus.

### 2.5 Statistical analysis

According to the formula of the Cochrane Toolkit for Systematic Evaluation: within-group difference of Mean = post-intervention in a group of Mean—baseline in a group of Mean, S^2^ = S_1_^2^ + S_2_^2^–2 × R × S_1_ × S_2_, where S is the value of within-group standard deviation, S_1_ is the post-intervention standard deviation, S_2_ is the baseline standard deviation, and R is a constant taken as 0.4 or 0.5 (8). For studies assessing outcome indicators, only data from the initial and final interventions were used if there were multiple intervention time points.

In this study, we employed Stata 16.0 and Revman 5.4 software for conducting Meta-analysis, using mean difference (MD) or standardized mean difference (SMD) as the indicator of the effect size, When the units of the outcome indicators were identical, MD was chosen as the effect indicator; when the units of the outcome indicators were different, SMD was chosen as the effect indicator (8), and the results were considered statistically significant when *p* ≤ 0.05 for the effect size. To assess inter-study heterogeneity, we performed quantitative analyses using the *I*^2^ statistic; heterogeneity was considered low if *p* > 0.1 and *I*^2^ < 50%, at which point a fixed-effects model was selected for analysis. Sensitivity analyses were conducted using individual study-by-study exclusion methods. In the case of high heterogeneity, we then used the random effects model. In addition, to ensure the credibility of the findings, we also used funnel plots and Egger’s test for the number of studies greater than 10 to assess potential publication bias, with a test level of α = 0.05.

## 3 Results

### 3.1 Study selection

**Figure 1:**
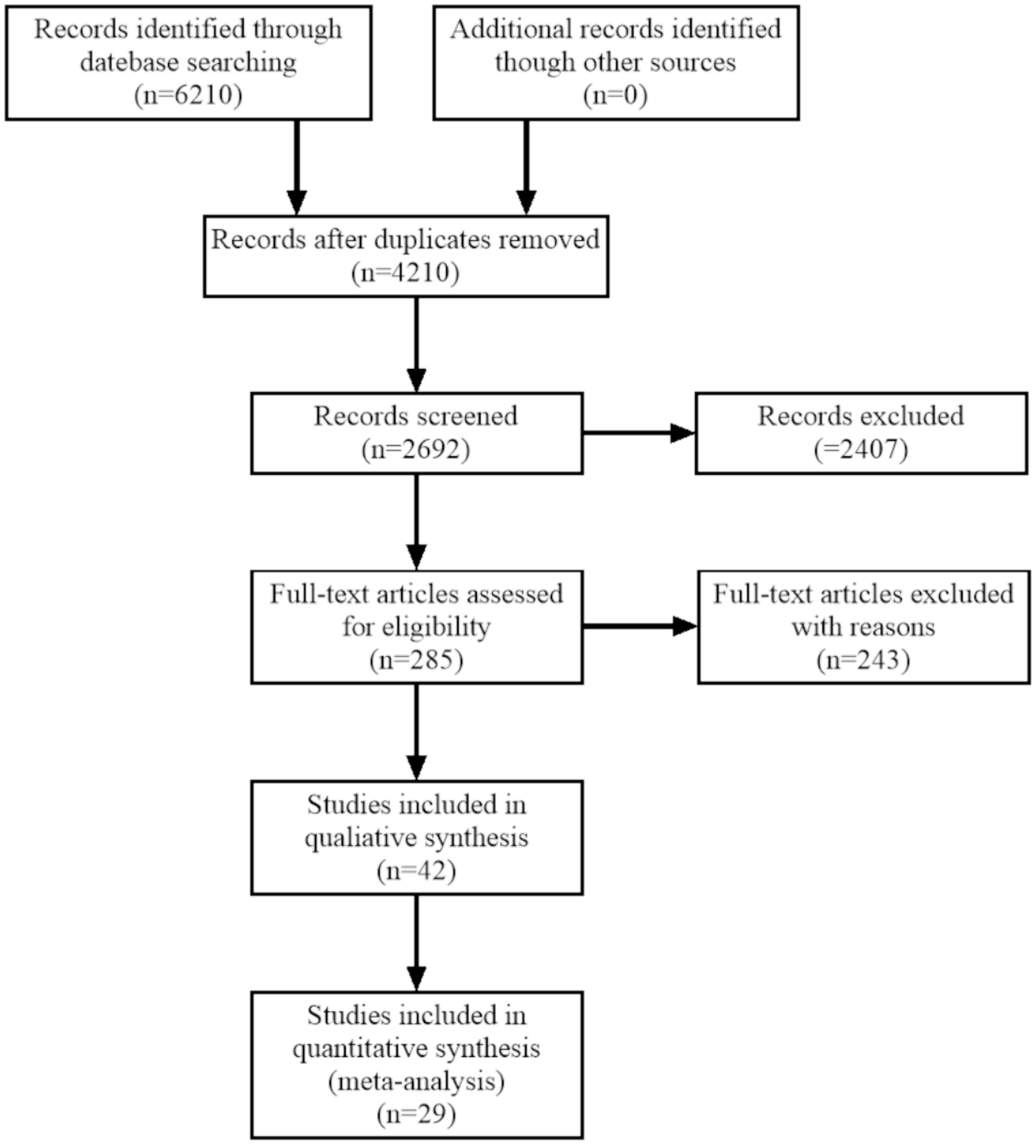
Flow chart of literature retrieval.

After the search, a total of 6,210 pieces of literature were identified. A total of 29 eligible studies were screened for inclusion through an initial review of titles and abstracts, as well as further full-text scrutiny, including 12 in Chinese and 17 in English. The screening process of literature is detailed in Figure 1.

### 3.2 Study characteristics

The 29 included studies comprised a total of 2,195 children and adolescents with overweight/obesity, and all of these studies categorized the participants into experimental and control groups. Table 2 displays the fundamental attributes of the studies selected.

**Table 2:** General characteristics of the included studies.

First author	Age (years)	Sample ratio (T/C)	Period (week)	Sessions/week	Session length (min)	Intervention details	Outcome measures
Ahmadi (2020) (9)	14.41±2.64	29/27	8	3	30	WU(10 min) + HIIT + RT(3 sets, 12 reps/set)	①②⑥⑦⑧⑨
Bruyndonckx (2015) (10)	12~18	33/28	40	3	30	AE + RT + Swimming Lessons	①⑤⑧⑨
Chae (2010) (11)	10.5±3.42	19/19	12	2	90	WU(10min) + AE, RT(70min, 6~7 of METs) + CD(10min)	①③④⑤⑥⑦⑧⑨
Chen (2015) (12)	14.3±3.31	15/15	8	3	55~60	WU(5~10 min) + AE(20 min, 60% of VO_2max_) + RT(20 min, 8~12 reps of 1-RM, 2~3 reps/set, 1~2 min/reps of rest between exercise bouts, 3~5 min/set rest between exercise bouts)	①③④⑤
Cheng (2012) (13)	13~14	30/30	8	2	70	AE+RT+Game	①③⑤⑥⑦⑧⑨⑫
Duft (2020) (14)	14.58±1.05	19/18	12	3	60	WU(5 min) + RT(6 sets, 6~10 reps/set) + AE(50%~85% of VO_2peak_)	①②④⑦⑩⑫⑬
Farpour-Lambert (2009) (15)	9±1.5	22/22	12	3	60	AE(30 min) + RT(30 min, 2~3 sets, 10~15 reps/set)	①⑥⑧⑩⑪⑫
Grace (2021) (16)	14.22±0.71	22/19	10	2	40	WU(5 min) + RT(10 min, Individualized progressive training, 5 different exercises each stage, 5 sets, 30 s/set, 14~17 of RPE) + AE(20 min) + CD(5 min)	①⑥⑧⑨⑩⑫⑬
Gui (2019) (17)	9~11	34/35	12	3	80	Traction(5 min) + WU(5min)+ AE(40min, 50%~70% of HRR) + RT(20 min, 8~12 reps of RM) + CD(10 min)	①
Jeon (2013) (18)	—	8-7	12	2	80~85	AE(30~35min, 55%~75% of MHR) + RT(9 sets, 70% of 1-RM)	①②⑥⑧⑩⑫⑬
Jia (2022) (19)	16.65±1.04	24/24	12	4	45	WU(8min)+ 2 reps/week AE(60%~75% of MHR), 2 reps RT + CD(5 min)	①④
Kim (2022) (20)	16~18	13/13	12	3	50	WU(5 min) + AE(20 min, 55%~75% of MHR) + RT(20min, 11~12 of RPE, 8~15 reps/set) + CD(5 min)	②④⑥⑦⑨
Li (2008) (21)	19.4±1.2	12-12	12	4	60	WU(5 min) + AE(30 min, 60%~80% of MHR) + RT(20 min, 60%~80% of the MHR) + CD(5 min)	①⑩⑪
Liu (2008) (22)	12.4±0.6	25/24	36	4~5	60~90	12-week, AE(5 reps/week, 60~90min, 3~7 of METs) + RT(2 reps/week, 60 min, 3~7 of METs); 24-week, AE(4 reps/week, 30~40 min, 3~7 of METs) + RT(2 reps/week, 20 min, 3~7 of METs)	⑥⑦⑧⑨
Lopes (2016) (23)	14.5±1.14	17/16	12	3	60	AE(50%~85% of VO_2peak_) + RT(6~10 reps of RM)	①④⑥⑦⑩⑫⑬
Loureiro (2020) (24)	11.09±2.06	21/49	12	3	60	—	①②④⑤
Martínez (2008) (25)	9.42±0.68	579/465	24	3	90	WU(15 min) + AE(60 min)+ RT(15 min)	①④
Pan (26)	13.97±0.82	15/17	12	6	180	AE(100min in three batches, 57%~67% of MHR) + RT(60min, 70%~80% of 1-RM, 4 sets, 6~12 reps/set)	①③④
Park (2012) (27)	12.15±0.11	14/15	12	3	80	WU(10min) + AE(30min, 50%~70% of HRR) + RT(7 sets, 8~12reps/set, 60% of 1-RM)	①②⑤⑥⑦⑧⑨⑪⑫⑬
Sung (2002) (28)	8~11	41/41	6	—	75	WU(10 min) + RT(20 min, 20 training stations, 10 reps/set of 1-RM) + AE(10min) + Agility training(10 min) + CD(5 min)	①③⑥⑦⑧⑨
Wang (2018) (29)	11~14	25/25	12	3	60	AE(15 min) + Fun Athletics(25 min) + RT(10 min) + CD(10 min), [AE，Fun Athletics and RT: 120~140 reps/min of the heart rates]	①③④
Wang (2007) (30)	8.68±1.78	12-12	8	6	120	AE(70min) + RT(50 min, 60%~70% of MHR)	①②⑥⑦⑫
Wang (2005) (31)	13.57±0.47	27/18	12	4~5	60	WU(5~10 min) + Exercise(40~50 min, 60%~65% of VO_2max_) + CD(5 min)	①②③④
Wong (2008) (32)	14.05±1.31	12-12	12	2	45~62	WU(7~10 min) + AE (25 min, 65%~85% of MHR, divided into 3~5 aerobic stations, each station 5 ~10 minutes) + RT(20~35min, 65%~85% of MHR, divided into 4~7 resistance stations and the number of exercise circuits gradually increased from 1 to 3 sets, repeated 8~25 times, each station 1~3min) + CD(7~10 min)	①③⑥⑦⑧⑨⑫
Wu (2020) (33)	—	9-9	16	3	70	WU(5 min) + AE(50 min, 60%~69% of MHR) + RT(10 min, 60%~69% of MHR) + CD(5 min)	①②⑥⑦⑧⑨⑫
Xiang (2019) (34)	12.89±1.51	18/18	6	6	300	AE(150~180 min, 50%~90% of MHR) + RT(60~90 min, 3~4 sets, 12~15 reps, 40%~50% of Maximum power)	①
Zehsaz (2016) (35)	10.55±0.92	16/16	16	2	45~60	WU(7~10 min) + AE + RT(65%~85% of MHR) + CD(7~10 min)	①②③④⑥⑦⑧⑨⑩⑫⑬
Zhang (2018) (36)	10.81±0.72	11-7	8	3	60	WU(5min) + AE(40min) + RT(15min, 60%~69% of MHR)	①②⑥⑦⑧⑨⑩⑫⑬
Zhen (2010) (37)	12.07±0.81	30/30	48	3	60	Initial stage: AE(10 min) + RT(25 min) + Endurance training(10 min, 110~130reps/min of the heart rates), Second stage: Endurance training, third stage: Aerobic endurance training	①④

T, Experimental group; C, Control group; —, unreported; AE, Aerobic exercise; RT, Resistance training; WU, Warm-up; CD, Cool-down; MHR, Maximum heart rate; HRR, Heart rate reserve; HIIT, High-intensity interval training; METs, Metabolic equivalent; RPE, Perceived exertion rating. ①BMI; ②WC; ③FM; ④BF%; ⑤VO_2max_; ⑥TG; ⑦TC; ⑧HDL-C; ⑨LDL-C; ⑩HOMA-IR; ⑪hs-CRP; ⑫FPG; ⑬INS.

### 3.3 Study quality assessment

The risk of bias assessment showed that of the 23 RCTs included, six described specific randomized sequence methods, six performed allocation concealment, and one used blinding, and all of the included literature had data completeness and were free of other biases and selective reporting. The quality of the literature of the 6 included non-randomized controlled trials was of high quality.

### 3.4 Meta-analysis

#### 3.4.1 Comparative analysis of BMI interventions

**Figure 2:**
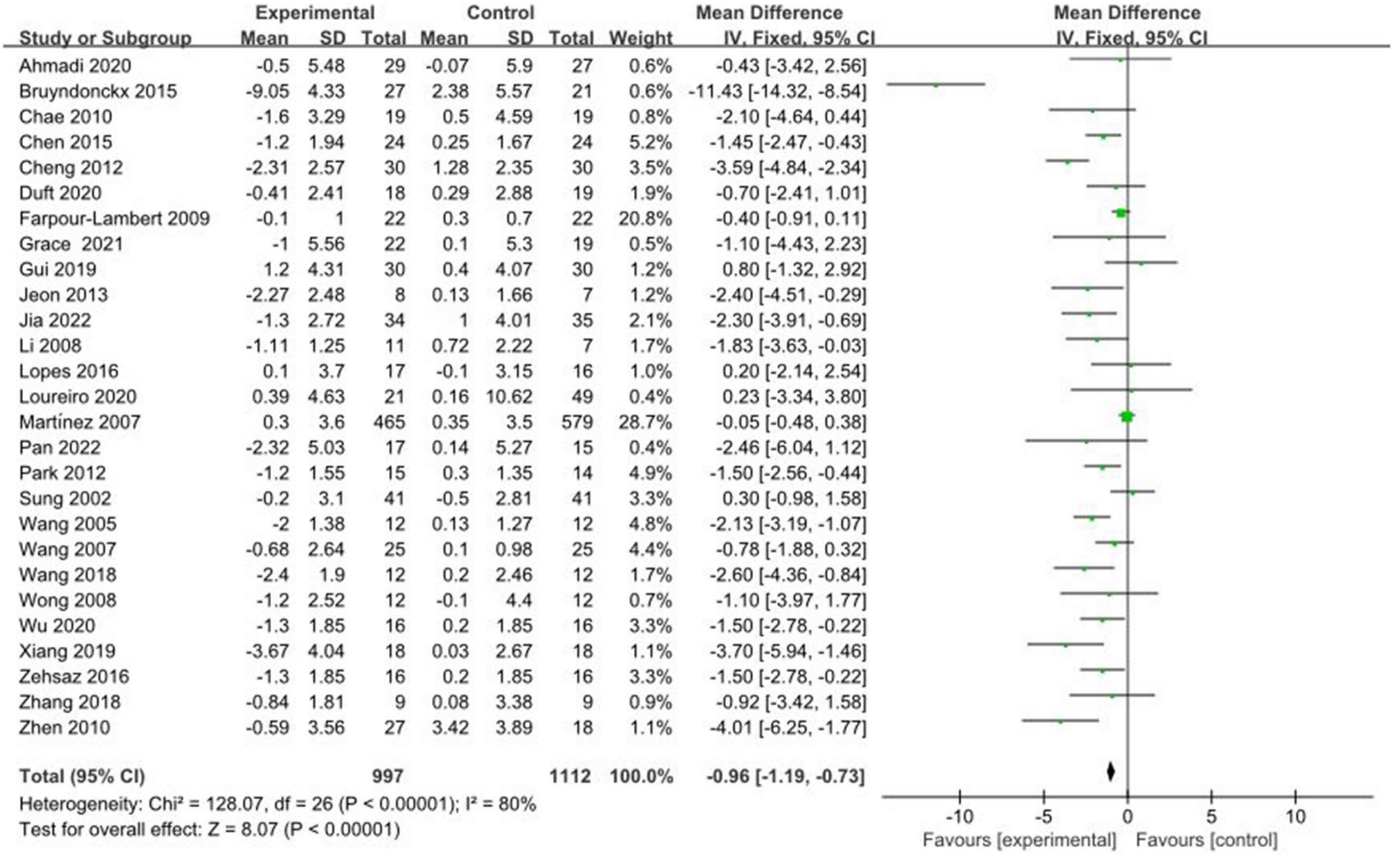
Forest plot of comparison: BMI.

A total of 26 studies reported AE + RT to evaluate BMI in children and adolescents with overweight/obesity. The result of a meta-analysis using a random-effects model showed that the BMI of the trial group was significantly lower than that of the control group after the intervention [MD = −0.96, 95%CI: −1.19, −0.73, *p* < 0.01] (Figure 2).

#### 3.4.2 Comparative analysis of WC interventions

**Figure 3:**
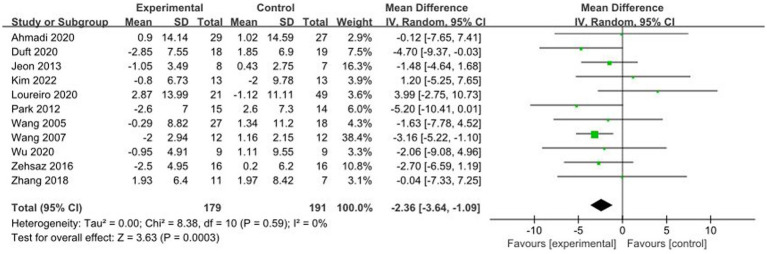
Forest plot of comparison: WC.

A total of 11 studies reported AE + RT to evaluate WC in children and adolescents with overweight/obesity. The result of a meta-analysis using a fixed-effects model showed that WC was significantly lower in the experimental group than in the control group after the intervention [MD = −2.36, 95%CI: −3.64, −1.09, *p* < 0.01] (Figure 3).

#### 3.4.3 Comparative analysis of FM interventions

**Figure 4:**
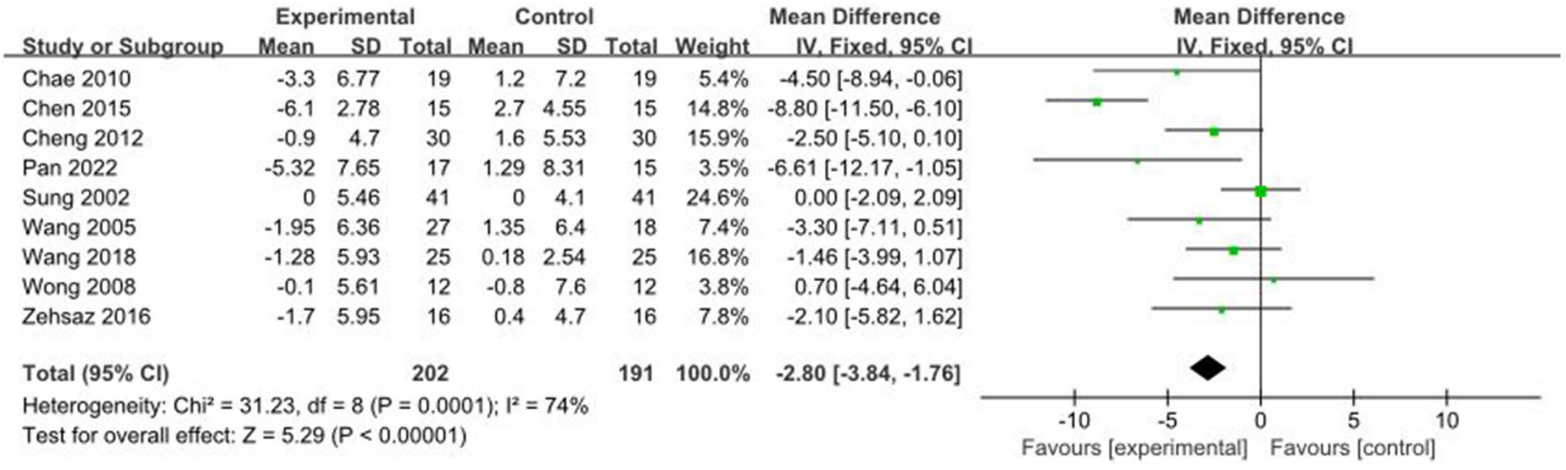
Forest plot of comparison: FM.

A total of 9 studies reported AE + RT to evaluate FM in children and adolescents with overweight/obesity. The result of a meta-analysis using a random-effects model showed that FM was significantly lower in the trial group than in the control group after the intervention [MD = −2.80, 95%CI: −3.84, −1.76, *p* < 0.01] (Figure 4).

#### 3.4.4 Comparative analysis of BF% interventions

**Figure 5:**
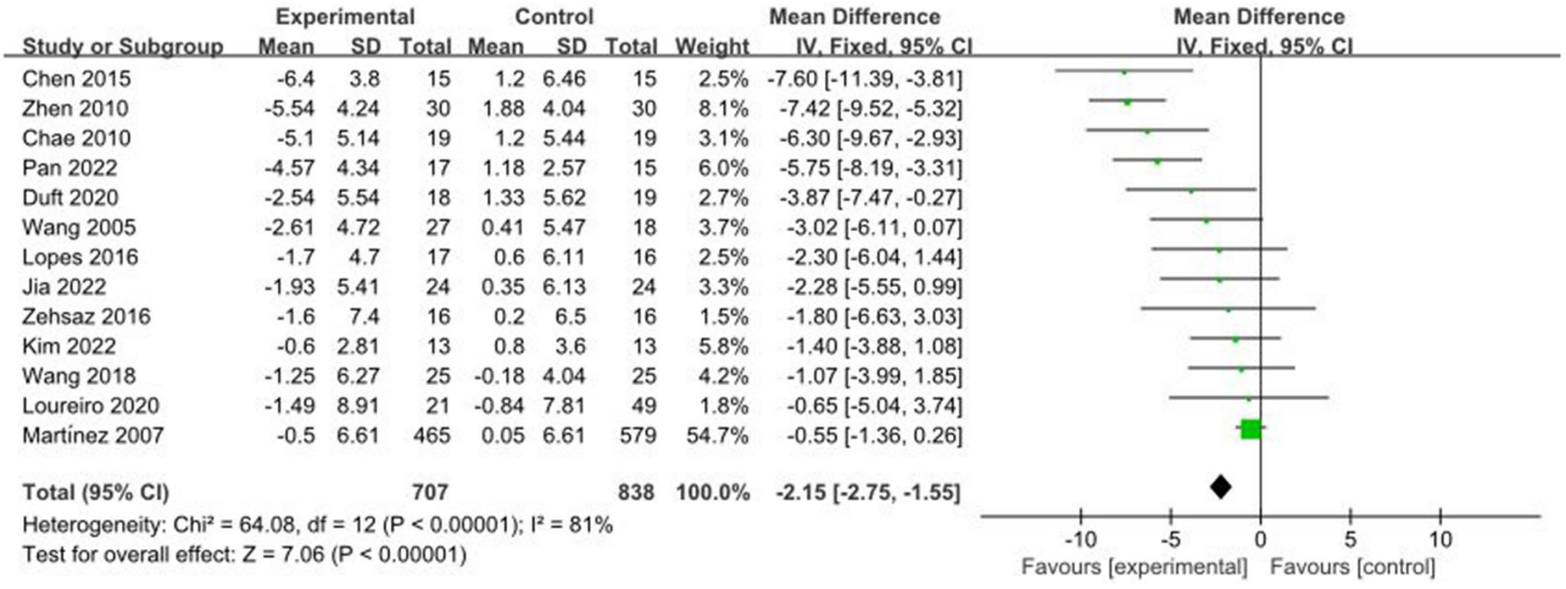
Forest plot of comparison: BF%.

A total of 13 studies reported AE + RT to evaluate BF% in children and adolescents with overweight/obesity. The result of a meta-analysis using a random-effects model showed that BF% in the trial group was significantly lower than that of the control group after the interventions [MD = −2.15, 95%CI: −2.75, −1.55, *p* < 0.01] (Figure 5).

#### 3.4.5 Comparative analysis of VO_2max_ interventions

**Figure 6:**
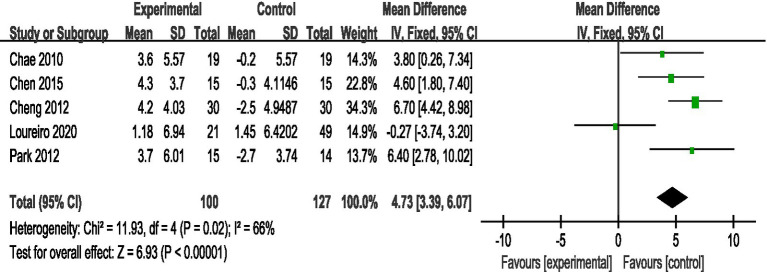
Forest plot of comparison: VO_2max_.

A total of 5 studies reported AE + RT to evaluate VO_2max_ in children and adolescents with overweight/obesity. The result of a meta-analysis using a random-effects model showed that the VO_2max_ in the trial group was significantly higher than that in the control group after the intervention [MD = 4.73, 95%CI: 3.39, 6.07, *p* < 0.01] (Figure 6).

#### 3.4.6 Comparative analysis of TG interventions

**Figure 7:**
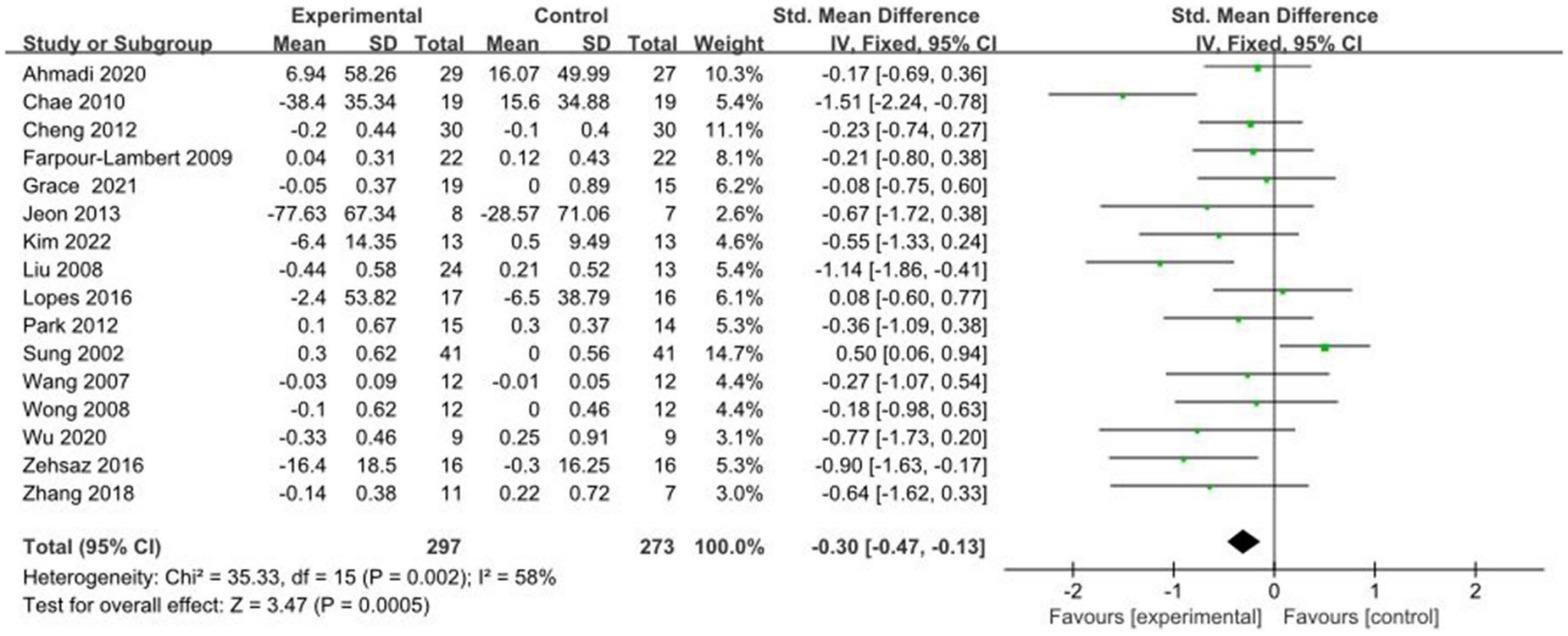
Forest plot of comparison: TG.

A total of 16 studies reported AE + RT to evaluate TG in children and adolescents with overweight/obesity. The result of a meta-analysis using a random-effects model showed that TG was significantly lower in the trial group than in the control group after the intervention [SMD = −0.30, 95%CI: −0.47, −0.13, *p* < 0.01] (Figure 7).

#### 3.4.7 Comparative analysis of TC interventions

**Figure 8:**
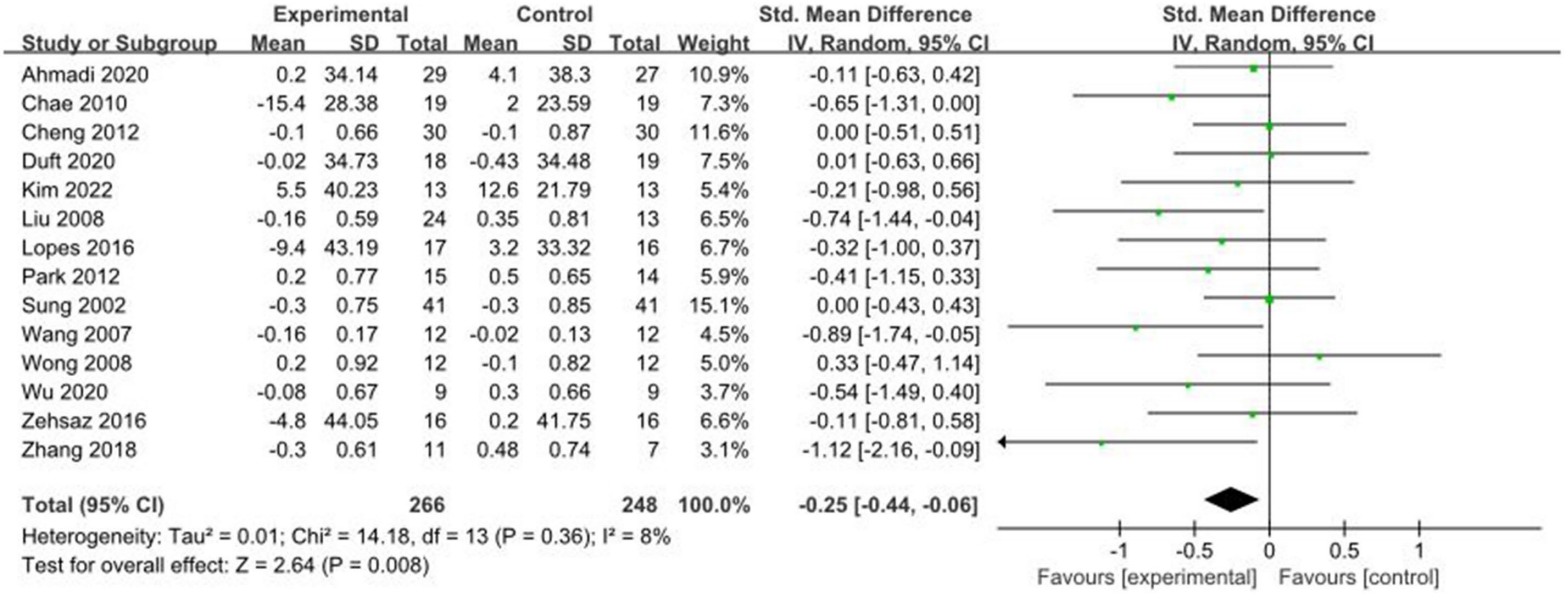
Forest plot of comparison: TC.

A total of 14 studies reported AE + RT to evaluate TC in children and adolescents with overweight/obesity. The result of a meta-analysis using a random-effects model showed that TC was significantly lower in the trial group than in the control group after the intervention [SMD = −0.25, 95%CI: −0.44, −0.06, *p* < 0.01] (Figure 8).

#### 3.4.8 Comparative analysis of HDL-C interventions

**Figure 9:**
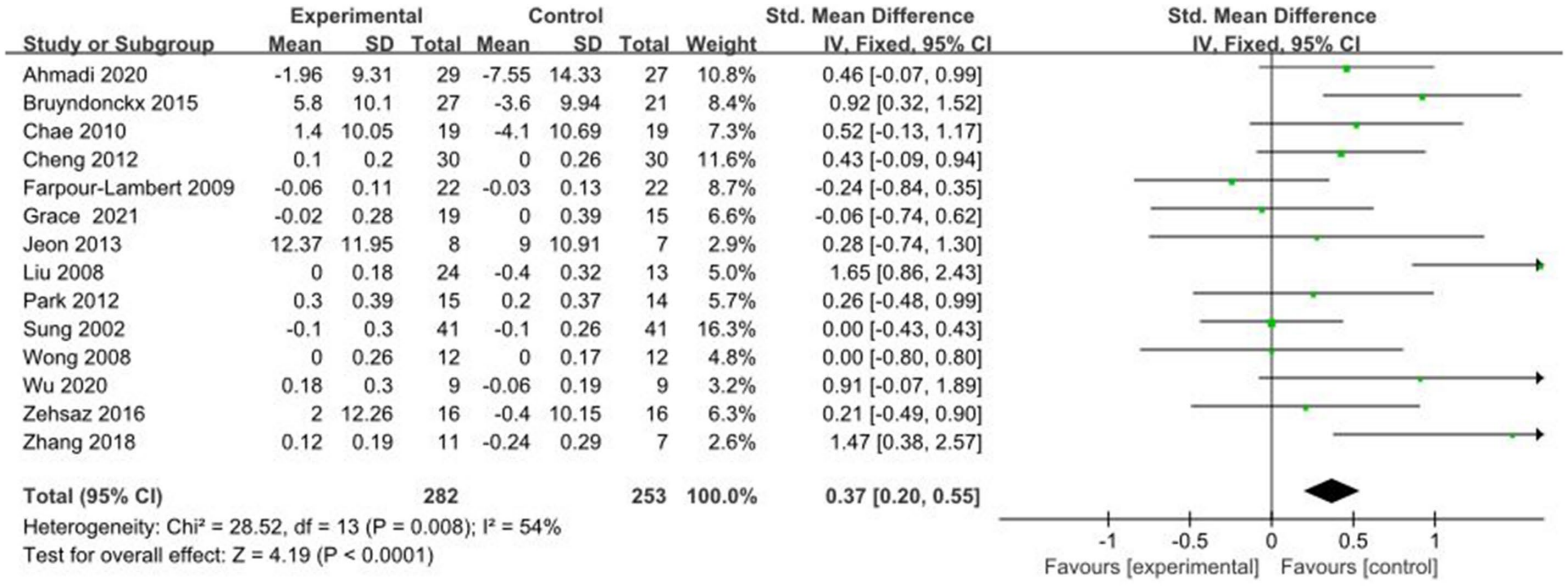
Forest plot of comparison: HDL-C.

A total of 14 studies reported AE + RT to evaluate HDL-C in children and adolescents with overweight/obesity. The result of a meta-analysis using a random-effects model showed that HDL-C was significantly higher in the experimental group than in the control group after the intervention [SMD = 0.37, 95%CI: 0.20, 0.55, *p* < 0.01] (Figure 9).

#### 3.4.9 Comparative analysis of LDL-C interventions

**Figure 10:**
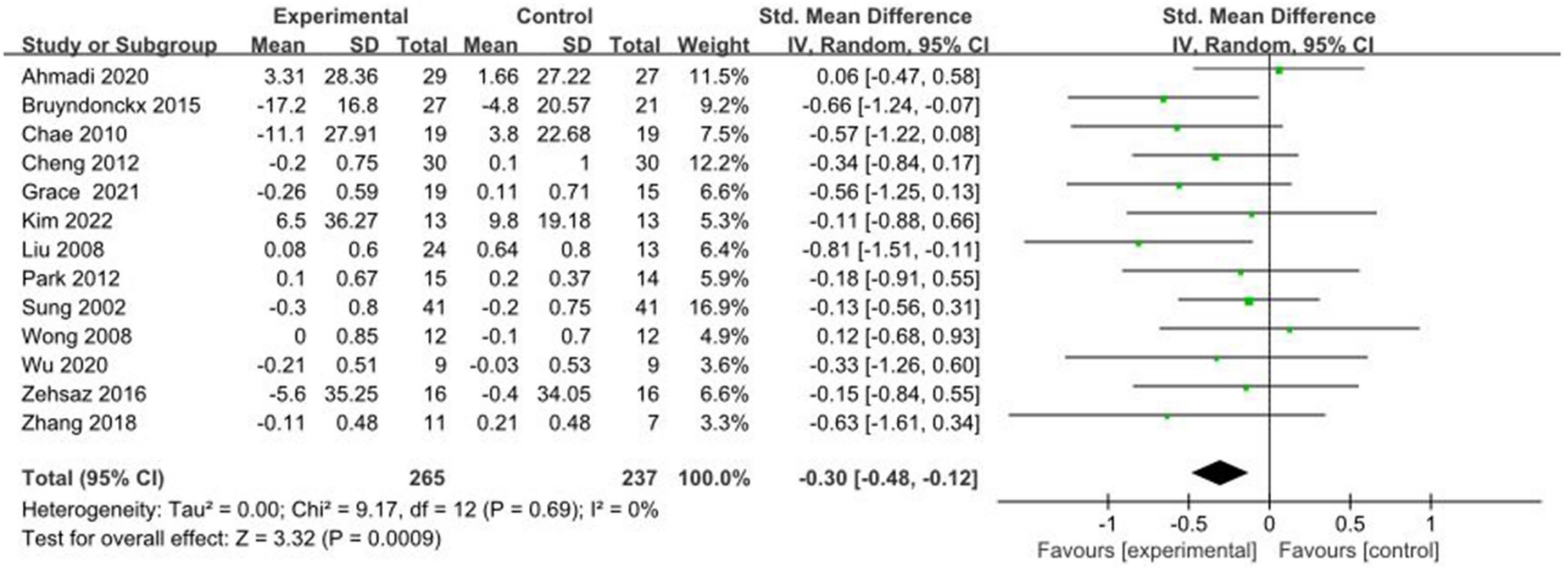
Forest plot of comparison: LDL-C.

A total of 13 studies reported AE + RT to evaluate LDL-C in children and adolescents with overweight/obesity. The result of a meta-analysis using a fixed-effects model showed that LDL-C was significantly lower in the experimental group than in the control group after the intervention [SMD = −0.30, 95%CI: −0.48, −0.12, *p* < 0.01] (Figure 10).

#### 3.4.10 Comparative analysis of HOMA-IR interventions

**Figure 11:**
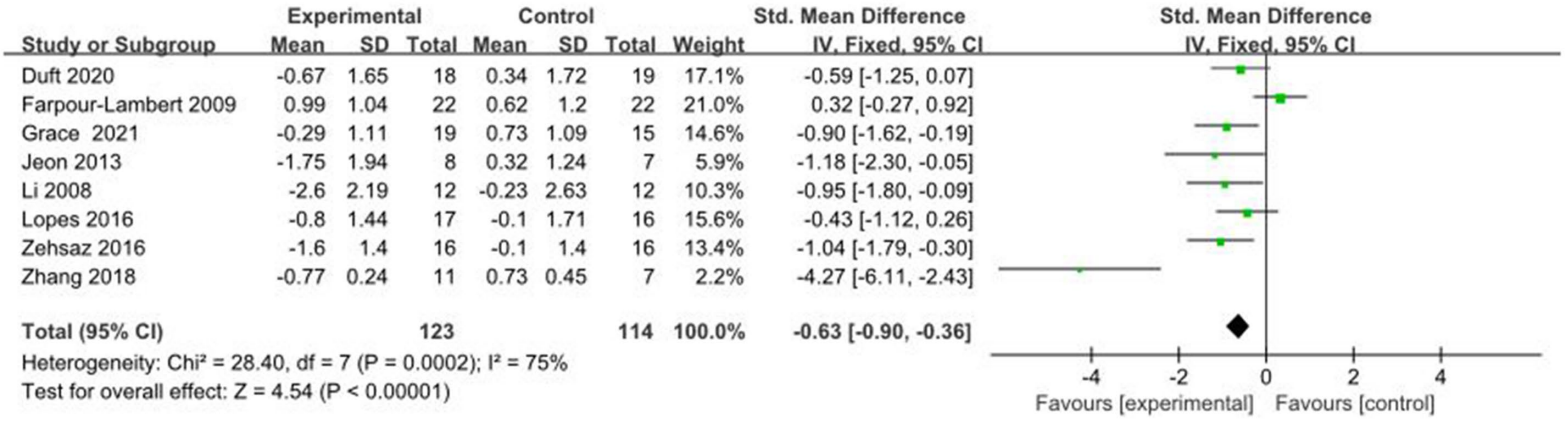
Forest plot of comparison: HOMA-IR.

A total of 8 studies reported AE + RT to evaluate HOMA-IR in children and adolescents with overweight/obesity. The result of a meta-analysisusing a random-effects model showed that HOMA-IR was significantly lower in the test group than in the control group after the intervention [SMD = −0.63, 95%CI: −0.90, −0.36, *p* < 0.01] (Figure 11).

#### 3.4.11 Comparative analysis of hs-CPR interventions

**Figure 12:**
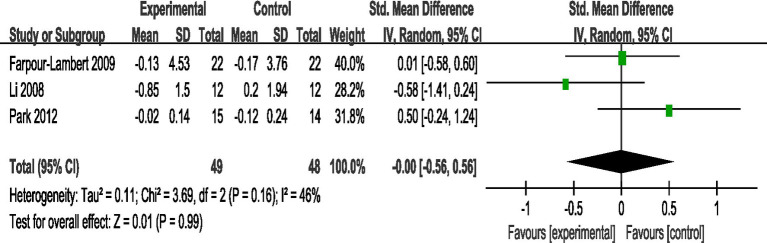
Forest plot of comparison: hs-CRP.

A total of 3 studies reported AE + RT to evaluate hs-CRP in children and adolescents with overweight/obesity. The result of a meta-analysis using a fixed-effects model showed that hs-CRP was not significant in the test group after the intervention [SMD = −0.00, 95%CI: −0.56, 0.56, *p* = 0.99] (Figure 12).

#### 3.4.12 Comparative analysis of FPG interventions

**Figure 13:**
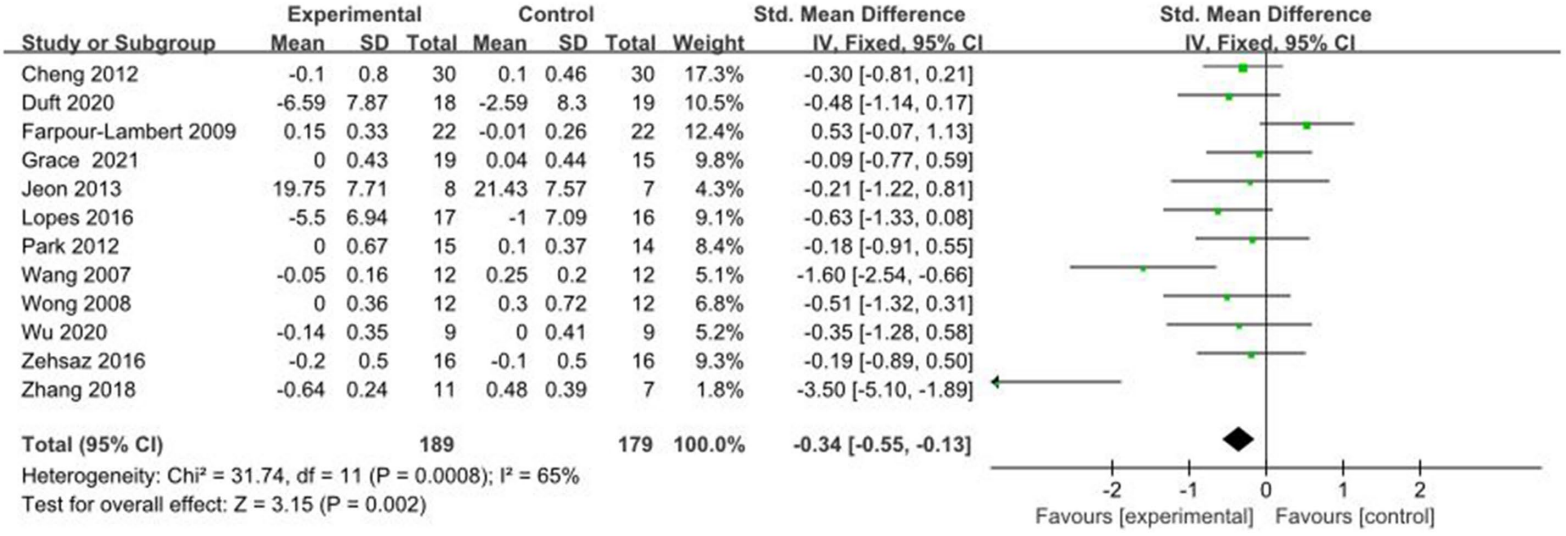
Forest plot of comparison: FPG.

A total of 12 studies reported AE + RT to evaluate FPG in children and adolescents with overweight/obesity. The result of a meta-analysis using a random-effects model showed that FPG was significantly lower in the experimental group than in the control group after the intervention [SMD = −0.34, 95%CI: −0.55, −0.13, *p* < 0.01] (Figure 13).

#### 3.4.13 Comparative analysis of INS interventions

**Figure 14:**
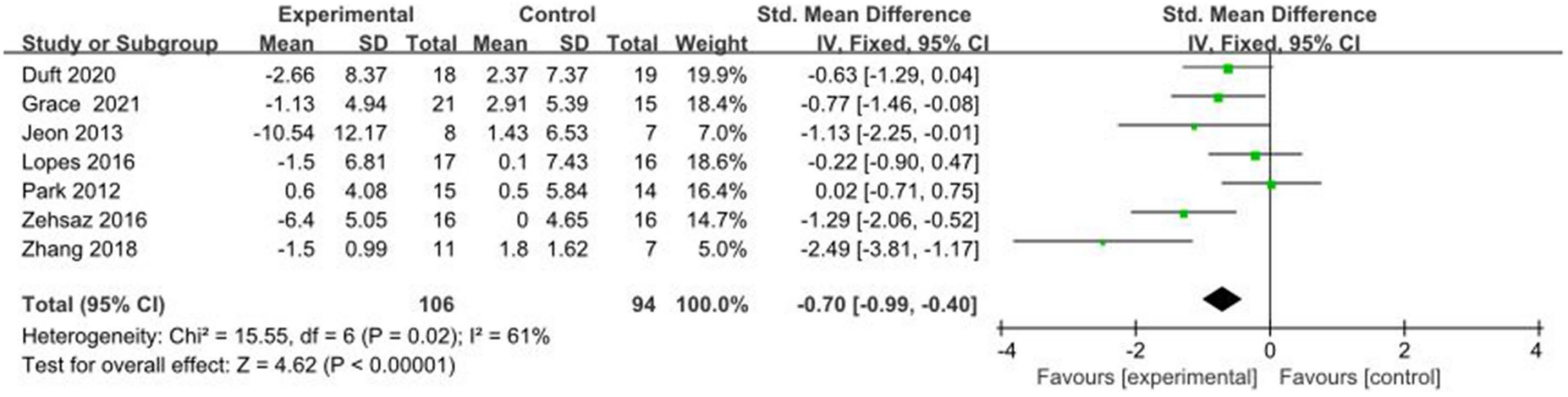
Forest plot of comparison: INS.

A total of 7 studies reported AE + RT to evaluate INS in children and adolescents with overweight/obesity. The result of a meta-analysis using a random-effects model showed that INS was significantly higher in the experimental group than in the control group after the intervention [SMD = −0.70, 95%CI: −0.99, −0.40, *p* < 0.01] (Figure 14).

### 3.5 Results of subgroup analyses

#### 3.5.1 Analysis of BMI subgroups

Differences in training parameters may be an important factor influencing fitness, therefore subgroup analyses were performed based on the training period, training frequency, and training time. The results showed that a training period of ≥12 weeks [MD = −1.51, 95%CI: −1.93, −1.08, *p* < 0.01], a training frequency of ≥3 times/week [WMD = −1.78, 95%CI: −2.35, −1.21, *p* < 0.01], and a duration of each training session of ≥60 min intervention was the best [MD = −1.49, 95%CI: −2.02, −0.97, *p* < 0.01] (Table 3).

**Table 3:** Subgroup analysis of BMI.

Outcome measures	No. of studies	Heterogeneity test results		Effects models	Meta-analysis results		
		*p*	*I* ^2^		Effect size indicator	95%CI	*p*
Intervention period							
≥12 weeks	15	0.44	1%	Fixed	MD	−1.51 [−1.93, −1.08]	<0.01
<12 weeks	8	0.01	60%	Fixed	MD	−1.27 [−1.90, −0.64]	<0.01
Intervention frequency							
≥3times	14	0.19	25%	Random	MD	−1.78 [−2.35, −1.21]	<0.01
<3times	6	0.38	6%	Random	MD	−1.28 [−2.16, −0.40]	<0.01
Intervention time							
≥60 min	20	0.03	40%	Random	MD	−1.49 [−2.02, −0.97]	<0.01
<60 min	3	0.81	0%	Random	MD	−1.32 [−2.25, −0.39]	<0.01

#### 3.5.2 Analysis of TG subgroups

The results showed that a training period of ≥12 weeks [SMD = −0.66, 95%CI: −1.01, −0.31, *p* < 0.01], a training frequency of ≥3 times/week [SMD = −0.30, 95%CI: −0.58, −0.02, *p* < 0.05], and a duration of each training session of ≥60 min intervention was optimal [SMD = −0.27, 95%CI: −0.47, −0.06, *p* = 0.01] (Table 4).

**Table 4:** Subgroup analysis of TG.

Outcome measures	No. of studies	Heterogeneity test results		Effects models	Meta-analysis results		
		*p*	*I* ^2^		Effect size indicator	95%CI	*p*
Intervention period							
≥12 weeks	9	0.08	43%	Random	SMD	−0.66 [−1.01, −0.31]	<0.01
<12 weeks	6	0.14	40%	Random	SMD	−0.06 [−0.38, 0.26]	0.72
Intervention frequency							
≥3times	7	0.78	0%	Fixed	SMD	−0.30 [−0.58, −0.02]	<0.05
<3times	5	0.03	62%	Fixed	SMD	−0.46 [−0.77, −0.15]	<0.01
Intervention time							
≥60 min	11	0.01	66%	Fixed	SMD	−0.27 [−0.47, −0.06]	0.01
<60 min	3	0.64	0%	Fixed	SMD	−0.22 [−0.59, 0.14]	0.23

#### 3.5.3 Analysis of HOMA-IR subgroups

The results showed that the best intervention effect was achieved with a training period of ≥12 weeks [SMD = −0.76, 95%CI: −1.11, −0.42, *p* < 0.01] and a training frequency of ≥3 sessions/week [SMD = −1.21, 95%CI: −2.20, −0.22, *p* < 0.05] (Table 5).

**Table 5:** Subgroup analysis of HOMA-IR.

Outcome measures	No. of studies	Heterogeneity test results		Effects models	Meta-analysis results		
		*p*	*I* ^2^		Effect size indicator	95%CI	*p*
Intervention period							
≥12 weeks	5	0.65	0%	Fixed	SMD	−0.76 [−1.11, −0.42]	<0.01
<12 weeks	2	<0.01	91%	Fixed	SMD	−1.34 [−2.01, −0.68]	<0.01
Intervention frequency							
≥3times	4	<0.01	80%	Fixed	SMD	−1.21 [−2.20, −0.22]	<0.05
<3times	3	0.92	0%	Fixed	SMD	−1.01 [−1.48, −0.54]	0.01

#### 3.5.4 Analysis of FPG subgroups

The results showed that the best intervention effect was achieved with a training period of ≥12 weeks [SMD = −0.38, 95%CI: −0.67, −0.09, *p* = 0.01] and a training frequency of ≥3 times/week [SMD = −0.92, 95%CI: −1.58, −0.25, *p* < 0.01] (Table 6).

**Table 6:** Subgroup analysis of FPG.

Outcome measures	No. of studies	Heterogeneity test results		Effects models	Meta-analysis results		
		*p*	*I* ^2^		Effect size indicator	95%CI	*p*
Intervention period							
≥12 weeks	7	0.97	0%	Fixed	SMD	−0.38 [−0.67, −0.09]	0.01
<12 weeks	4	<0.01	85%	Fixed	SMD	−0.60 [−0.96, −0.24]	<0.01
Intervention frequency							
≥3times	6	<0.01	72%	Fixed	SMD	−0.92 [−1.58, −0.25]	<0.01
<3times	5	0.95	0%	Fixed	SMD	−0.26 [−0.57, 0.05]	0.01

### 3.6 Sensitivity analysis

Sensitivity analyses conducted on the included studies revealed no significant directional changes in the combined effect sizes upon the individual exclusion of studies. This indicates a robustness in the results of the meta-analyses. Due to space limitations, detailed results of the sensitivity analyses are available upon request from the corresponding author.

### 3.7 Publication bias

**Figure 15:**
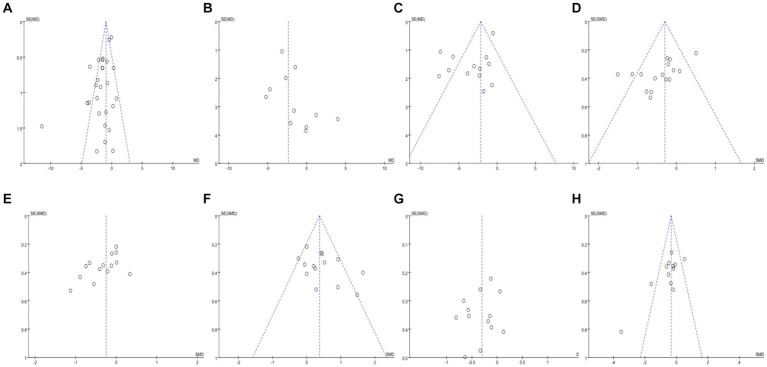
Funnel plots for different outcome indicators.

The funnel plots for studies involving BMI, WC, BF%, TG, TC, HDL-C, LDL-C, and FPG were generated and subjected to Egger’s bias test (Figure 15).

The distribution of the BMI funnel plot was roughly uneven, and Egger’s test yielded a *Pr* > |*z*| = 0.00, which is less than 0.05, indicating the presence of publication bias (Figure 15A). The WC funnel plot distribution was also roughly uneven, but Egger’s test resulted in a *Pr* > |*z*| = 0.15, which is greater than 0.05, suggesting no publication bias (Figure 15B). The BF% funnel plot distribution was roughly uneven, with Egger’s test showing a *Pr* > |*z*| = 0.00, less than 0.05, indicating publication bias (Figure 15C). The TG funnel plot distribution was roughly uneven, and Egger’s test showed a *Pr* > |*z*| value less than 0.05, indicating the presence of publication bias (Figure 15D). The TC funnel plot distribution was also roughly uneven, with Egger’s test yielding a *Pr* > |*z*| = 0.02, less than 0.05, indicating publication bias (Figure 15E). The HDL-C funnel plot distribution was roughly uneven, and Egger’s test showed a *Pr* > |*z*| = 0.02, less than 0.05, indicating publication bias (Figure 15F). The LDL-C funnel plot distribution was roughly even, and Egger’s test resulted in a *Pr* > |*z*| = 0.44, greater than 0.05, suggesting no publication bias (Figure 15G). Finally, the FPG funnel plot distribution was roughly uneven, with Egger’s test showing a *Pr* > |*z*| = 0.00, less than 0.05, indicating publication bias (Figure 15H).

## 4 Discussion

In the present study, AE + RT was found to be effective in improving several health indicators (except hs-CRP) in children and adolescents with overweight/obesity. This finding aligns with a prior study (38), suggesting a potential synergistic effect of AE + RT on diverse health parameters. Long-term and regular aerobic exercise reduces fat accumulation, leads to a decrease in plasma insulin, and improves overall physical function. Resistance exercise sustains energy expenditure after training and promotes muscle tissue production. The scientific arrangement of the two types of exercise contributes to the overall improvement of physical condition (39). The observed high heterogeneity in certain indicators may stem from disparities in training variables and variations in the proportions of AE and RT combinations, leading to notable discrepancies in the outcomes.

HDL-C is commonly associated with a diminished risk of cardiovascular disease due to its role in scavenging excess cholesterol from arterial walls and transporting it to the liver for metabolism and elimination. Data from 14 pieces of literature in this study showed that AE + RT increased HDL-C in children, and the literature was moderately heterogeneous (*I*^2^ = 54%). These findings deviate from the results reported in the meta-analysis conducted by Tianhao Chen (40) and other researchers, potentially attributed to the inclusion of studies focusing solely on aerobic exercise in their analyses, thus influencing the outcomes. Additionally, some scholars (41) suggested that HDL-C only improves when exercise intensity or duration reaches a certain threshold.

In our investigation, no significant difference was observed in hs-CRP levels, which contrasts with the findings reported by Jingqi Liu (42) et al. Their study revealed that both AT and AT+RT were effective in reducing CRP levels in adolescents with obesity, with AT+RT demonstrating superiority over AT alone. However, two out of the three studies included in the present study did not show a significant effect, which may be due to the differences in baseline indices, and differences in the duration and methodology of the intervention. Further research is warranted to elucidate the precise reasons underlying these inconsistencies.

Four key outcome metrics (BMI, TG, HOMA-IR, and FPG) were selected for the relevant subgroup analyses. These metrics were chosen to provide comprehensive information for a comprehensive assessment of health problems in children and adolescents with overweight/obesity. They encompass aspects of body mass, cardiovascular health, metabolic health, and insulin metabolism, collectively providing valuable insight into the health status and associated risks of children and adolescents with overweight/obesity.

In the subgroup analyses of the four indicators, studies with training cycles exceeding 18 weeks were excluded to align with practical considerations such as current teaching cycles and school schedules. Upon analyzing the BMI and TG subgroups, it was observed that children and adolescents with overweight/obesity benefited significantly from a training cycle of ≥12 weeks comprising more than three sessions per week, each session lasting over 60 min, resulting in notable improvements in BMI and TG levels. Therefore, it is recommended to adjust the ratio of AE to RT based on these training parameters to obtain a more stable and reliable effect.

When subgroup analyses of HOMA-IR and FPG were performed, training time subgroup analyses were not performed due to the limited number of studies in the training time subgroup. The results of the studies showed that training cycles up to 12 weeks showed larger effect sizes for both HOMA-IR and FPG, but the heterogeneity of the relevant literature was high (*I*^2^ > 70%). This discrepancy may be due to the higher sensitivity of human blood glucose levels to AE and the fact that the effect of interventions on glucose metabolism may differ when the ratio of AE to RT is different (43).

This study is subject to several limitations: (1) Dietary interventions and health education were not systematically excluded from the included literature. (2) Subgroup analyses based on age and gender among children and adolescents were not conducted. (3) The study did not conduct comparative analyses with either aerobic or resistance exercise alone, nor did it consider the potential influence of factors such as region, ethnicity, and lifestyle habits. (4) The synergy between aerobic and resistance exercises could not be quantified.

## 5 Conclusion

In summary, future studies should strive to incorporate detailed considerations of the characteristics of both aerobic and resistance exercises, while also taking into account factors such as age, body mass index, and psychological traits of children and adolescents with overweight/obesity. Based on the current evidence, the recommended exercise prescription is at least three sessions of more than 60 min per week for 12 weeks or more for better health benefits.

## Data availability statement

The original contributions presented in the study are included in the article/supplementary material, further inquiries can be directed to the corresponding author.

## Author contributions

XL: Conceptualization, Data curation, Writing – original draft, Writing – review & editing. QL: Writing – review & editing. FL: Methodology, Software, Supervision, Validation, Writing – review & editing. DZ: Investigation, Supervision, Writing – review & editing.
